# Determinants and Factors of Physical Activity After Oncology Treatments (DEFACTO) in Metropolitan France: Protocol of a Mixed Methods Study and Intervention

**DOI:** 10.2196/52274

**Published:** 2024-05-16

**Authors:** Albane Aumaitre, Rémi Gagnayre, Aude-Marie Foucaut

**Affiliations:** 1 Health Educations and Promotion Laboratory, LEPS, UR 3412 Sorbonne Paris North University Villetaneuse France; 2 Sports Science Department Sorbonne Paris North University Bobigny France

**Keywords:** socioecological model, mixed methods, cancer survivorship, physical activity, sedentary behavior, individualized health education program, feasibility

## Abstract

**Background:**

While the scientific community widely recognizes the benefits of physical activity (PA) in oncology supportive care, cancer survivors who have undergone chemo- or radio-immunotherapy treatments struggle to meet PA recommendations. This underscores the importance of identifying factors influencing active lifestyle adoption and maintenance and proposing a multilevel model (micro-, meso-, and macrolevel) to better understand facilitators and barriers. Currently, no socioecological model explains an active lifestyle in the posttreatment phase of breast, colorectal, prostate, and lung cancers.

**Objective:**

The objective is to identify factors influencing an active lifestyle in cancer survivorship and assess the feasibility of an individualized program targeting an active lifestyle. The objectives will be addressed in 3 stages. Stage 1 aims to elucidate factors associated with the active lifestyle of cancer survivors. Stage 2 involves developing an explanatory model based on previously identified factors to create a tailored health education program for an active lifestyle after oncology treatments. Stage 3 aims to evaluate the feasibility and potential effects of this personalized health education program after its national implementation.

**Methods:**

First, the exploration of factors influencing PA (stage 1) will be based on a mixed methods approach, using an explanatory sequential design and multilevel analysis. The quantitative phase involves completing a questionnaire from a socioecological perspective. Subsequently, a subset of respondents will engage in semistructured interviews to aid in interpreting the quantitative results. This phase aims to construct a model of the factors influencing an active lifestyle and develop an individualized 12-week program based on our earlier findings (stage 2). In stage 3, we will implement our multicenter, multimodal program for 150 physically inactive and sedentary cancer survivors across metropolitan France. Program feasibility will be evaluated. Measured PA level by connected device and multidimensional variables such as declared PA and sedentary behaviors, PA readiness, motivation, PA preferences, PA knowledge and skills, and barriers and facilitators will be assessed before and during the program and 52 weeks afterward.

**Results:**

The institutional review board approved the mixed methods study (phase 1) in April 2020, and the intervention (phase 3) was approved in March 2022. Recruitment and data collection commenced in April 2022, with intervention implementation concluded in May 2023. Data collection and full analysis are expected to be finalized by July 2024.

**Conclusions:**

The Determinants and Factors of Physical Activity After Oncology Treatments (DEFACTO) study seeks to enhance our understanding, within our socioecological model, of factors influencing an active lifestyle among cancer survivors and to assess whether a tailored intervention based on this model can support an active lifestyle.

**Trial Registration:**

ClinicalTrials.gov NCT05354882; https://www.clinicaltrials.gov/study/NCT05354882

**International Registered Report Identifier (IRRID):**

DERR1-10.2196/52274

## Introduction

### Background

The beneficial effects of physical activity (PA) in oncology have been widely demonstrated by the scientific community [1]. This nonpharmacological intervention [2] is now recognized as supportive care in oncology in France [3], given its efficacy in improving quality of life [4], reducing cancer-related fatigue [5], and decreasing the relative risk of cancer recurrence [6]. A high level of PA (ie, 600 to 900 metabolic equivalents of task [METs] as h/week) postdiagnosis appeared to be relatively protective against cancer-specific mortality in breast, colorectal, and prostate cancers, as well as all-cause mortality in breast, colorectal, lung, and prostate cancers [7].

Despite the documented benefits of PA and the risks associated with sedentary behavior (SB), defined as any waking behavior in a sitting or lying position, many adult cancer survivors who have completed chemo-radio-immunotherapy treatments remain insufficiently active and may even engage in SB [8]. While advancements in cancer management have led to a 20% increase in patient survival [9], adverse effects persist long after treatment completion, with a gradual decline observed over 10 to 15 years [10]. The posttreatment period, where the end of treatments marks a break within the “clinical pathway,” is particularly crucial to understand. Health promotion is often overlooked during follow-up medical checkups [11]. Furthermore, individuals who have completed treatment must readjust their daily routines to balance returning to work with family responsibilities while rebuilding their sense of self [12]. Consequently, cancer survivors embark on the “care pathway,” wherein they grapple with managing their health behaviors [13], including an active lifestyle. For instance, upon returning to work, individuals frequently report feeling more tired, making it challenging to strike the right balance between rest and PA [14].

### Previous Work

Studies delineate personal factors (eg, self-efficacy, PA history, and motivation) and environmental factors (eg, equipment accessibility and health care system) implicated in adherence to PA during and after cancer treatment [15,16]. French researchers have proposed a conceptual framework for physio-psychological mechanisms—3H (hypodynamia, hypokinesia, or hypoxia) syndrome—[17], while others aim to propose a psychosocial model [18]. Socioecological approaches to health education interventions in PA [19] offer a framework for identifying the intricate interrelations between cancer survivors and their environment. While acknowledging the importance of individual factors in behavioral change, such as stage of change, processes of change, decisional balance, self-efficacy, and knowledge, socioecological approaches also encompass influences at organizational, environmental, and policy levels. These approaches constitute one of the main pillars of promoting health-enhancing PA [20]. Thus, there is a need to enhance our understanding of active lifestyle in oncology, as highlighted in the French National Cancer Institute report on PA in 2017 [4] and other international reports [21]. To our knowledge, there is no study examining factors influencing PA and SB among breast, prostate, colorectal, or lung cancer survivors, using the transtheoretical model (TTM) [22] as a framework and structuring collected data within a socioecological model [19].

Exploration and intervention targeting factors associated with an active lifestyle—clinical, psychological, cognitive, behavioral, social, or even linked to the clinical pathway and the geographical environment—can provide insights into the complex behaviors of PA and SB, as well as potential influences on them.

### Aims

**Figure 1:**
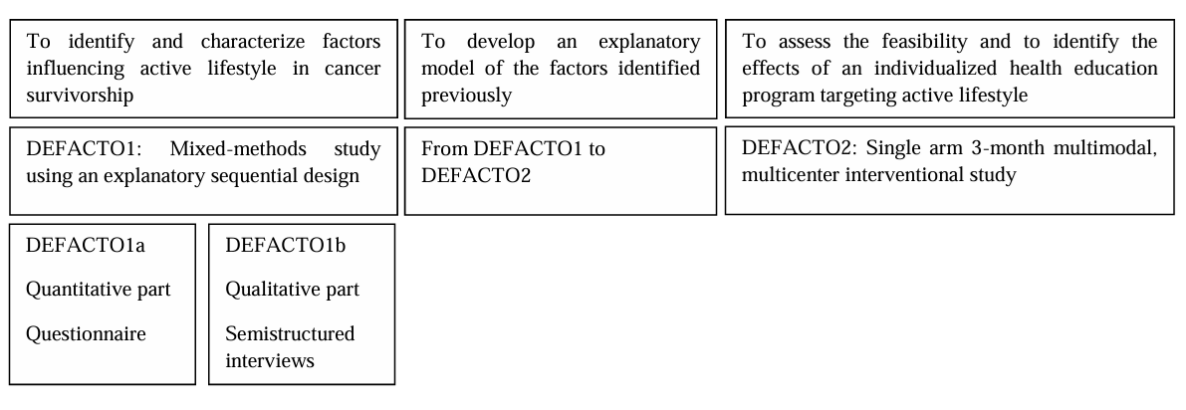
Design of the Determinants and Factors of Physical Activity After Oncology Treatments (DEFACTO) Study.

The 3 phases of the Determinants and Factors of Physical Activity After Oncology Treatments (DEFACTO) study are described in Figure 1.

The first phase seeks to identify and characterize the factors that positively and negatively influence PA and SB among breast, colorectal, prostate, and lung cancer survivors, ranging from 3 weeks to 20 years after chemo- or radio-immunotherapy treatment. This phase of the study (DEFACTO1) will use a mixed methods approach. Participants’ “lifestyle profiles” will be delineated based on their PA and SB levels: (1) high PA and low SB, (2) high PA and high SB, (3) low to moderate PA and low SB, and (4) low to moderate PA and high SB [23,24].

The second phase aims to develop an explanatory model [19] for categorizing the previously identified factors.

The third phase is dedicated to assessing the feasibility and potential effects of a multimodal, individually tailored health education program designed in accordance with the model. The program will be specifically tailored for physically inactive and sedentary cancer survivors. A verification of the preestablished model will also be conducted.

## Methods

### Phase 1: Exploratory Study Using a Mixed Methods Approach

The mixed methods approach of DEFACTO1 adheres to the Good Reporting of A Mixed Methods Study recommendations [25]. The sampling, collection, and analysis of quantitative and qualitative data, as well as the integration of phases [26], are outlined below.

An explanatory sequential design is used, beginning with a quantitative phase, followed by a qualitative research phase. Given the multifaceted nature of an active lifestyle, the use of mixed methods allows for a comprehensive exploration of this complex topic [27]. Quantitative data obtained from a self-reported questionnaire are used to develop a semistructured interview guide. Additionally, qualitative data derived from semistructured interviews aids in interpreting and understanding the quantitative data [28].

### Quantitative Phase (DEFACTO1a)

The aim is to identify factors influencing an active lifestyle among cancer survivors. A questionnaire titled “DEFACTO questionnaire” was developed between September 2019 and January 2020 from validated questionnaires on factors influencing PA practice, ranging from the microlevel to the macrolevel [29].

#### Recruitment

##### Overview

The French UNICANCER group on supportive care, the French League Against Cancer, and patient associations are invited to participate in quantitative data collection by distributing the questionnaire to cancer survivors (digital or paper survey). To ensure the most representative sample and avoid solely including cancer survivors from hospitals, and associations, information is shared on social networks (eg, Facebook [Meta], LinkedIn [Microsoft Corporation], and X [formerly known as Twitter]). Information and consent forms are provided to participants in both digital and paper formats.

##### Inclusion Criteria

Participants in the quantitative study must be aged 18 years or older; diagnosed with breast, prostate, colorectal, or lung cancer (stage 1 to 3 cancer, primary, second and relapse included); have completed chemo- or radio-immunotherapy treatment within the past 3 weeks and up to 20 years; be able to read, comprehend, and respond to a questionnaire in French; and have signed the informed consent form for the study.

##### Exclusion Criteria

Participants are excluded if diagnosed with metastatic cancer; residing outside mainland France to maintain focus on the French context; having an absolute medical contraindication to PA, as this is a known barrier to engagement for these cancer survivors and cannot be intentionally changed through lifestyle interventions; being pregnant or breastfeeding; being under guardianship, curatorship, or deprived of liberty.

Considering the median age at diagnosis for all cancer types combined in France, projected to be 68 years for women and 70 years for men [30], we aimed to target the broadest possible population during the initial phase of the study. The study encompasses cancer survivors ranging from 3 weeks to 20 years posttreatment, recognizing this time frame as the period during which late effects of treatments may still be experienced [31]. The 3-week threshold corresponds to the point at which cancer survivors realizes that the end of treatments does not indicate a return to life as it was before diagnosis [32]. The study focuses on the 4 most common French cancers—breast, prostate, colorectal, and lung—for which the benefits of PA are well established [4,30].

#### Data Collection and Assessments

The self-administered DEFACTO questionnaire [29] was developed by integrating validated questionnaires and various other validated scales. Estimated PA level through different domains (ie, occupational PA, leisure-time PA, and transport-related PA) and estimated SB are collected by the self-reported Global Physical Activity Questionnaire (GPAQ) [33]. Data collected through the GPAQ are the PA level (in MET-min/week), the time spent in moderate to high intensity PA in minutes per week (min/week), and the time spent in SB in minutes per day (min/day). Variables from the TTM—stages of change, processes of change, decisional balance, and self-efficacy—are collected with the following validated questionnaires: Stages of Change of Exercise Behavior Scale [34,35], exercise processes of change [36], decisional balance for exercise [37], and Exercise Confidence Survey [38]. Types of PA motivation are collected through the Echelle de Motivation envers l’Activité Physique en contexte de Santé (EMAPS), a motivation scale toward health-oriented PA [39].

A subcategory of health literacy level is collected using a single question concerning the need for help to understand health information [40]. The idea is not to assess health literacy levels but to identify cancer survivors with special needs according to this single question. Quality of life is assessed by the Short-Form 12 [41]. Stereotypes related to the benefits of exercise are explored through specific items extracted from the Cancer Beliefs and Exercise Scale (items 5, 15, 20, and 30) [42]. PA barriers already identified in the general population are collected with the “Barriers to Being Active Quiz” [43]. This 21-item tool examines 7 areas: (1) lack of time, (2) social influences, (3) lack of energy, (4) lack of willingness, (5) fear of getting hurt, (6) lack of skill sets, and (7) lack of resources. A total score of 6 or above in any area indicates that this is an important barrier to PA for the respondent. The Barriers to Being Active Quiz also gathers data concerning body image, pleasure felt during PA practice, the individual’s environment (eg, home space walkability, sport facilities, and equipment), and the availability of the entourage, all of which are variables identified within the theoretical framework as influencing PA participation [44,45]. It also explores the PA participation of social and family environments in 3 items (eg, opportunities to practice PA with a close circle, other commitments relating to family life, and other people’s perception of oneself).

#### Other Assessments

Finally, PA experiences, sociodemographics, anthropometrics, clinical data, and tobacco use are also collected. Cancer survivors complete the whole DEFACTO questionnaire once. All variables are gathered from it.

##### PA Experience

Respondents are asked a series of open-ended and closed-ended questions to ascertain whether they have benefited from an adapted physical activity (APA) during their cancer treatment. This includes inquiries into the modalities of the program (eg, type of supervision, location, and frequency of meetings) and the satisfaction level, rated from 0 (not satisfied at all) to 10 (very satisfied).

##### Sociodemographic Data

Sociodemographic data include age (in years); sex (male or female); education level (>college, college, high school, or <high school); economic category (<EUR 600, EUR 600-EUR1200, EUR 1200-EUR 2000, EUR 2000-EUR 3000, and >EUR 3000 per month [A currency exchange rate of EUR 1=US $1.08 is applicable]); professional category (without employment, in sick leave, student, retired, or worker); household type (alone, single-parent family, couple without children, couple with children, or other configuration); accommodation (flat or house) and area of residence (urban or rural); and subscription to health insurance (yes or no).

##### Anthropometric Data

Weight (kg) and height (cm) are declared by the respondent. BMI (in kg/m²) is calculated, and cancer survivors are classified into normal weight (18.5 kg/m² and 24.9 kg/m²), overweight (25 kg/m² and 29.9 kg/m²), and obesity (>30 kg/m²) categories [46].

##### Clinical Data

Clinical data related to cancer and comorbidities are assessed using items of the French survey of cancer survivors 2 years after diagnosis Vie après le CANcer à 2 ans du diagnostic (VICAN2) [8]: date of diagnosis (month and year), cancer location, stage, treatments received, time-lapse since the end of treatments (month and year), the presence or not of a second cancer—new primary cancer or recurrence—and the presence of other pathologies and disabilities.

##### Tobacco Use

Tobacco use and consumption are assessed through a closed-ended question. Actual smokers are invited to complete the 2 items of the Fagerström questionnaire related to tobacco dependence level [47].

#### Data Analysis

Descriptive and correlation analyses will be conducted using SAS software (version 9.4; SAS Institute Inc) and SAS STAT software (version 15.3; SAS Institute Inc). Cancer survivors reaching or not a high PA level (≥3000 MET-min/week) [48] and having or not a high SB (≥5 h/day) [23,24] will be categorized into four lifestyle profiles [23] as follows: (1) high PA and low SB (≥3000 MET-min/week and sitting <5 h/day), (2) high PA and high SB (≥3000 MET-min/week and sitting ≥5 h/day), (3) low to moderate PA and low SB (<3000 MET-min/week and sitting <5 h/day), and (4) low to moderate PA and high SB (<3000 MET-min/week and sitting ≥5 h/day).

Descriptive analysis and multivariate logistic regression will analyze the association between the lifestyle profiles of cancer survivors and factors identified through the DEFACTO questionnaire. Descriptive and correlation analyses will use SAS (version 9.4) and the SAS STAT package (version 15.3), presenting descriptive data as frequencies, percentages, and mean (SD) for continuous variables. The Kruskal-Wallis test will compare the distributions of processes of change and PA barriers among lifestyle profiles. The final multiple ordinal logistic regression model will be defined through stepwise selection, examining associations between lifestyle profiles and various parameters from the DEFACTO questionnaire. The ordinal logistic regression will use cumulative probability. Results from the multiple ordinal logistic regression will be reported as odds ratios and 95% CIs, with significance levels set at *P*<.05.

### Qualitative Phase (DEFACTO1b)

An interview grid will be developed based on significant results from the DEFACTO1a study.

#### Recruitment

Quantitative study respondents who agreed were contacted for the qualitative study. Cancer survivors are selected according to their lifestyle profile.

#### Data Collection

Semistructured interviews are carried out by telephone due to the health context related to COVID-19. After patient approval, interviews are recorded and transcribed. According to the mixed methods’ design, interviews’ topics correspond to the significant results of the multivariate logistic regression from the quantitative study.

#### Data Analysis

Transcripts of the interviews will be qualitatively analyzed using the Nvivo software (version 1.5.1; QSR International). After the coding of data and the exploration of major themes and topics of the interviews, data interpretation shall be based on the interview grid. Qualitative data will complete quantitative data according to the mixed methods research design.

### Phase 2: Ecological Model of PA and SB Based on DEFACTO1

The Booth model provides a global vision of factors influencing the initiation and maintenance of an active lifestyle [19]. Other socioecological models of PA determinants in the overall population have also emerged [49-51], highlighting microscopic or individual factors (eg, psychological factors and beliefs), mesoscopic factors (eg, family, housing, and security), and macroscopic factors (eg, societal, political, and legislative influences) [19]. Compared to other socioecological models, the Booth model appears to be the most adaptable, with its design featuring nested circles that effectively capture the complexity of health behaviors across various environments. Additionally, the model facilitates an understanding of the connections between these environments. To our knowledge, this model has not been previously used to elucidate factors associated with an active lifestyle among cancer survivors in a French context.

The socioecological model will be completed with barriers and facilitators to active lifestyle identified during the DEFACTO1 study and significantly associated with lifestyle profiles of cancer survivors.

### Phase 3: Health Education Program (DEFACTO2)

#### Study Design

The DEFACTO2 intervention is a single-arm 3-month health education program, aligning with the typical duration of interventions in this field [52,53] and the anticipated time needed to establish habits during behavior changes [54]. This multimodal, multicenter intervention is neither randomized nor controlled. Its purpose is to assess the feasibility of implementing an individualized program; further research is required to evaluate the effectiveness of DEFACTO2 in relation to variations in PA and SB.

#### Inclusion Criteria

Eligible participants are aged between 18 and 75 years old to avoid geriatric oncology [55] and to ensure population homogeneity in the feasibility study. They must be survivors of primary nonmetastatic breast, lung, prostate or colorectal cancer (stage 1 to 3, including primary, second cancer, and relapse), have completed chemo-radio-immunotherapy treatments within the past 3 weeks to 20 years, have no contraindications for individual discovery session of APA, be willing to commit to the DEFACTO2 3-month program and 12-month follow-up, be physically inactive and sedentary (ie, <150 min/week of moderate PA or <75 min/week of vigorous PA, with an average of 5 h/day or more of SB), be proficient in reading, understanding and speaking French, be capable of using the connected device (Garmin Vivosmart 4 [Garmin International Inc]) and performing basic operations (eg, pressing a button and charging the battery), possesses a smartphone or a computer to download the Garmin Connect app (Garmin International Inc), be able to walk unassisted (to prevent bias in connected device data), reside in France, and have signed the consent form.

DEFACTO2 targets physically inactive and sedentary cancer survivors, aiming to increase their PA to meet established guidelines [21,56]. A total of 145 participants are required to detect a change of 75 minutes of moderate-intensity PA per week between before and after the 3-month intervention, with an output of 90% and a 5% risk (2-sided). This calculation is based on a moderate intensity PA variation of 75 min/week according to the study of Lynch et al [57], an average moderate-intensity PA of 50.6 (SD 48.2) min/week in a subset of physically inactive cancer survivors from the DEFACTO1 study (not yet published), and a common SD of 140, considering the SD of moderate PA averages observed before and after the intervention in Lynch et al [57]’s study.

#### Recruitment

Information is disseminated among League Against Cancer committees, with calls for participation in the health education program sent through email. The 15 participating committees inform cancer survivors who meet the inclusion criteria, after which interested individuals contact the principal investigator through email or phone to schedule an appointment. Furthermore, calls for participation are distributed nationally to hospitals, patient associations, and social networks to maximize outreach to cancer survivors. Ultimately, we anticipated recruiting 10 volunteers per committee, each providing a certificate confirming no contraindication to PA and signing the consent form.

#### Intervention Design

The primary end point of the interventional phase of the DEFACTO2 study is the feasibility of a health education program aiming to increase moderate intensity PA by 75 min/week in physically inactive and sedentary cancer survivors. All participants undergo a 3-month program comprising an APA discovery session, group educational sessions, and 7 or 8 motivational interviews by telephone. The intervention duration follows the guidelines for exercise programs [56].

The APA individual discovery session will be supervised by a qualified APA professional trained in the DEFACTO approach with expertise in tailoring APA sessions. Participants will select an activity from a list of several APAs. The content of educational group sessions will be predefined by the research team, reflecting findings from the DEFACTO1 exploratory study. Only APA discovery sessions and educational sessions will be conducted in-person at the French League Against Cancer departmental committee sites.

A motivational interview is a kind of support strategy used here in a research context [58]. The motivational interview series begins with discussions on participants’ expectations and reasons for participation. Then, the content of motivational interviews is tailored based on participant’s responses to the DEFACTO questionnaire at inclusion (T_0_), focusing particularly on stage of change, processes of change [59], self-efficacy, motivation types, barriers, and facilitator data. Motivational interviews aim to provide practical support for implementing an active lifestyle, addressing individual needs such as balancing PA with work, managing family life, and discovering local PA options or programs, as well as financial support. As motivational interviews progress, an individualized education tool is developed to help participants visualize their barriers and facilitators within a socioecological model. The number of motivational interviews is determined based on the average of interventions in similar fields [60].

**Figure 2:**
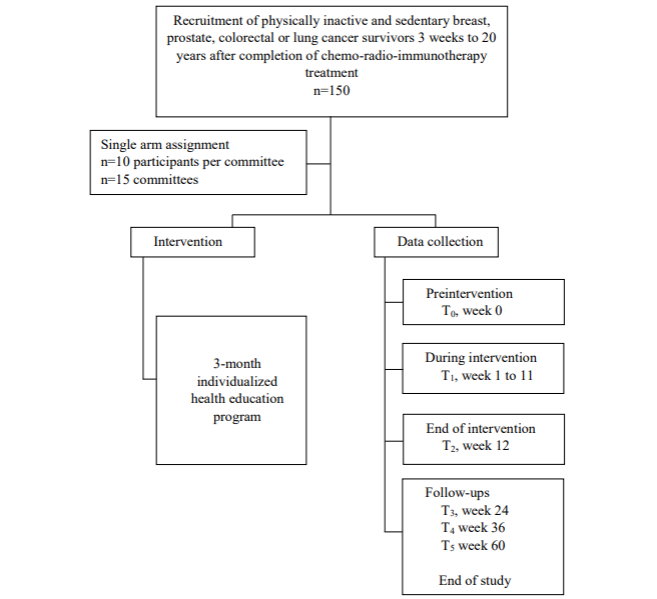
Flowchart for study design of Determinants and Factors of Physical Activity After Oncology Treatments (DEFACTO2).

Participants are followed up 12 months after the intervention, as shown in Figure 2.

All participants receive a Garmin Vivosmart 4 connected device to monitor PA and will retain the watch upon completion of the study.

#### Data Collection and Assessments

##### Overview

A total of 6 data collection periods are taking place (Table 1): before the intervention (T_0_), continuously during the 12-week intervention (T_1_), at the end of the 12-week intervention (T_2_), 3 months after the intervention for the first follow-up (T_3_), and at the 6- and 12-month follow-ups (T_4_ and T_5_, respectively). From T_2_ to T_5_, no specific instructions about PA or SB will be provided.

**Table 1:** Participant assessment and program evaluation Determinants and Factors of Physical Activity After Oncology Treatments (DEFACTO2).

Data collection period			Participant assessment and program evaluation
**Participant assessments**			
	T_0_: 1 week before intervention	PA^a^ barriers and facilitators, preferences, self-efficacy, motivation types, knowledge about PA and its link with cancer, subjective PA level and time spent in sedentary behaviors, quality of life (DEFACTO questionnaire) Objective PA level (Vivosmart 4)	
	T_1_: 12 weeks during DEFACTO intervention	Objective PA level (Vivomsart 4) Punctual and informal feedbacks on experience in the program Adherence to PA recommendations	
	T_2_: End of the 12th week of intervention	PA barriers and facilitators, preferences, self-efficacy, motivation types, knowledge about PA and its link with cancer, subjective PA level and time spent in sedentary behaviors, quality of life (DEFACTO questionnaire) Objective PA level (Vivosmart 4)	
	T_3_: 3-month postintervention follow-up	Objective PA level (Vivosmart 4)	
	T_4_: 6-month postintervention follow-up	Objective PA level (Vivosmart 4)	
	T_5_:12-month postintervention follow-up	Objective PA level (Vivosmart 4)	
**Program evaluation**			
	T_0_: 1 week before intervention	N/A^b^	
	T_1_: 12 weeks during DEFACTO intervention	Adherence to the program Adverse events declared by the participant	
	T_2_: End of the 12th week of intervention	Satisfaction and experience of participants regarding the individualized program, perception of the intervention’s usefulness Satisfaction of adapted physical activity professionals regarding the educational tools Fidelity of the intervention	
	T_3_: 3-month postintervention follow-up	N/A	
	T_4_: 6-month postintervention follow-up	N/A	
	T_5_:12-month postintervention follow-up	N/A	

^a^PA: physical activity.

^b^Not applicable.

##### DEFACTO Questionnaire

Participants will complete the questionnaire at T_0_ to develop the individualized education tool and tailor the intervention based on TTM processes of change, and PA barriers and facilitators. The questionnaire is completed again at T_2_ to identify modifications made during the intervention.

##### PA Preferences

Participants’ PA preferences will be collected at T_0_ through a brief interview for program individualization.

##### PA Level

Objective PA level will be measured using the Vivosmart 4 connected watch by Garmin. Variables required for each Garmin Connect account include sex, age (in years), weight (in kg), and height (in cm). These elements are required to calculate variables associated with PA measurement (explained in the Data Analysis section).

Average daily steps, distance traveled (km/day), floors climbed (number of floors climbed per day), heart rate (bpm), and cumulative duration of PA of moderate and vigorous intensities (min/week) will be collected over a 7-day period at T_0_, T_2,_ T_3_, T_4_, and T_5_. Data will be continuously collected from T_0_ to T_1_, covering a 12-week period [48].

Subjective PA level will be assessed using the GPAQ self-questionnaire [33] at T_0_ and T_2_.

##### Adherence

Adherence to the program will correspond to the number of sessions done regarding the planned sessions.

#### Program Evaluation

##### Satisfaction and Experience

At T_2_, overall satisfaction will be assessed, along with participants’ opinions on the individualized health education program. Open-ended questions will allow participants to express any aspects that may have been omitted and that would need to be incorporated into future interventions. Throughout the 3-month program, punctual and informal feedback will be collected, as well as potential adverse events reported by the participants.

##### Satisfaction of Qualified APA Professional

An interview will be conducted with all 15 professionals involved in the program at T_2_ to gather collective feedback on the overall program. The use and practicality of the communication medium used in educational group sessions will be specifically examined.

The feasibility of DEFACTO2 is evaluated based on criteria such as the recruitment process, and the capacity to target the intended participants, the appropriateness of procedures and measures in line with study objectives, and the study population. The acceptability and fidelity of the intervention are evaluated by including both open and closed questions in the DEFACTO questionnaire at T_2_. Subsequently, the resources and skills of the research team to implement the intervention are assessed, along with the potential effects of the program on an active lifestyle [61].

#### Data Analysis

Data analysis will use SAS software, summarizing the demographic and baseline characteristics of participants at T_0_. Continuous variables will be presented as means (SDs), while categorical variables will be reported as frequencies and percentages. Descriptive statistics will detail each component of the DEFACTO questionnaire.

For the primary outcome, both objective PA levels measured by Vivosmart 4 and subjective data will be considered. Usable data from continuous assessment, that is, when the connected watch is worn for at least 10 h/day and 4 days in a week, will be described through means (SDs). Following verification of data normality, comparative analysis will be conducted using 1-tailed student *t* test between objective PA levels at T_0_ and T_2_ to evaluate the primary outcome and between T_2_, T_3_, T_4_, and T_5_ to analyze PA maintenance after the 3-month program. The relationship between PA levels measured by Vivosmart 4 and estimated by the self-questionnaire (GPAQ) will be analyzed at T_0_ and T_2_.

A comparison will be conducted between T_0_ and T_2_ to assess the evolution of processes of change, self-efficacy, knowledge about PA and its link with cancer, motivation types, barriers, and facilitators to PA, declared PA and SB levels, and quality of life.

### Ethical Considerations

The Committee for the Protection of Persons (CPP) approved the DEFACTO1 mixed-methods study on April 3, 2020 (CPP South Mediterranean II, 2019-A03183-54). The DEFACTO2 intervention was approved on March 7, 2022 (CPP North West III, 2021-A01570-41) and registered on ClinicalTrials.gov on May 2, 2022 (NCT05354882). Free and informed consent was collected before any act related to research.

## Results

DEFACTO1a recruitment and data collection took place between April 2020 and January 2021. A total of 175 cancer survivors completed the DEFACTO questionnaire. From June 2021 to August 2021, semistructured interviews have been carried out with 18 participants. DEFACTO2 began in April 2022. The implementation of the intervention was concluded in May 2023, and data collection and full data analysis are expected to be completed by July 2024.

## Discussion

### Overview

DEFACTO uses a mixed methods approach with an explanatory sequential design and multilevel analysis. This approach aligns with implementation strategy recommendations and is informed by previous research on needs, barriers, and facilitators [62]. Our tailored and multimodal intervention, grounded in a socioecological model, aims to support physically inactive and sedentary cancer survivors in adopting and sustaining an active lifestyle. We anticipate this approach to be customizable, feasible, and applicable, fostering lasting behavior change with a broader impact on cancer survivors’ lifestyles. Future plans include implementing this program across the 97 departmental committees of the Metropolitan French League Against Cancer. This study will contribute to current research in the field of supportive care in cancer and in public health. This study addresses several gaps in current scientific studies, such as considering patients’ preferences (eg, type of PA proposed) and demographic and socioeconomic factors that remain underexplored and should be investigated more thoroughly [4,21]. The French context is particular in the field of active lifestyle promotion in the oncology care pathway, as it is not implemented in every country. For example, French physicians can prescribe exercise, and a new French decree allows cancer survivors to benefit from fitness and motivation assessment, including motivation interviews for PA (decree No. 2020-1665 of December 22, 2020, relating to the global care pathway after oncology care). Our program could complement current advances in the health care system, promoting an active lifestyle that can be incorporated into people’s daily lives.

### Expected Results

The implementation of an individualized active lifestyle program like DEFACTO2 may be influenced by various elements. These elements can either support or impede the program’s success. By examining these elements, we can pinpoint the circumstances conducive to implementing such interventions across different settings. This research protocol seeks to offer support based on a model for cancer survivors encountering challenges in embracing an active lifestyle through an individualized approach. This approach integrates an individual behavior change model with a broader examination of environmental influences on behavior. Additionally, it aims to equip professionals with practical tools to provide effective support to promote an active lifestyle among cancer survivors.

### Strengths and Limitations

This study has certain limitations. First, the GPAQ in the DEFACTO questionnaire gathers subjective PA and SB data, potentially leading to inaccuracies [63]. To address this, accelerometer-based PA measurements will be used in the third phase of the study.

Wearing a connected watch produces a motivating effect for PA, particularly linked to the “awareness” process of experiential change, with direct feedback from exercise [64], and associated with learning to self-measure [65]. The connected watch will serve as both an assessment tool and part of the support provided, as all participants will use it and frequently refer to it.

The DEFACTO questionnaire is not validated but uses validated questionnaires widely used in care pathways.

The committees of the French League Against Cancer have varying ongoing or upcoming projects, motivations, facilities, resources, organizations, and local policies. Ensuring feasibility across different League Against Cancer committees involves standardizing the proposed APA, communication methods, and participant instructions. A toolkit package and one-on-one training are mandatory for professionals involved in DEFACTO2 intervention study.

### Conclusions

The findings of this study will have significant implications for the feasibility of implementing tailored interventions promoting PA across the French metropolitan territory. Additionally, they will influence the implementation of PA initiatives in oncology care pathways worldwide. In addition to improving the active lifestyle among cancer survivors, such interventions have the potential to provide support to individuals facing difficulties after completing their oncology care pathway, such as better management of priorities and health behaviors.

## Abbreviations

APA: adapted physical activity
CPP: Committee for the Protection of Persons
DEFACTO: Determinants and Factors of Physical Activity After Oncology Treatments
EMAPS: Echelle de Motivation envers l’Activité Physique en contexte de Santé
GPAQ: Global Physical Activity Questionnaire
MET: metabolic equivalent of task
PA: physical activity
SB: sedentary behavior
TTM: transtheoretical model
VICAN2: Vie après le CANcer à 2 ans du diagnostic

## Data Availability

The data that will be generated during the study will be available from the corresponding author on request.
